# Bioinformatic Analysis of Crosstalk Between circRNA, miRNA, and Target Gene Network in NAFLD

**DOI:** 10.3389/fgene.2021.671523

**Published:** 2021-04-29

**Authors:** Cen Du, Lan Shen, Zhuoqi Ma, Jian Du, Shi Jin

**Affiliations:** ^1^The Fourth Affiliated Hospital of China Medical University, Shenyang, China; ^2^Shanghai Chest Hospital, Shanghai Jiaotong University, Shanghai, China

**Keywords:** NAFLD, non-coding RNAs, circRNA, miRNA, bioinformatic analysis

## Abstract

**Background:** The majority of chronic liver disease is caused by non-alcoholic fatty liver disease (NAFLD), which is one of the highly prevalent diseases worldwide. The current studies have found that non-coding RNA (ncRNA) plays an important role in the NAFLD, but few studies on circRNA. In this study, genes, microRNA (miRNA), and circular RNA (circRNA) associated with NAFLD were found by bioinformatic methods, bringing a novel perspective for the prevention and treatment of NAFLD.

**Methods:** Expression data of GSE63067 was acquired from Gene Expression Omnibus (GEO) database. The liver samples were collected from the people diagnosed with NAFLD or not. Differentially expressed genes (DEGs) were obtained from the steatosis vs. the control group and non-alcoholic steatohepatitis (NASH) vs. the control group using the GEO2R online tool. The overlapping genes remained for further functional enrichment analysis and protein-protein interaction network analysis. MiRNAs and circRNAs targeting these overlapping DEGs were predicted from the databases. Finally, the GSE134146 dataset was used to verify the expression of circRNA.

**Results:** In summary, 228 upregulated and 63 downregulated differential genes were selected. The top 10 biological processes and relative signaling pathways of the upregulated differential genes were obtained. Also, ten hub genes were performed in the Protein-protein interaction (PPI) network. One hundred thirty-nine miRNAs and 902 circRNAs were forecast for the differential genes by the database. Ultimately, the crosstalk between hsa_circ_0000313, miR-6512-3p, and *PEG10* was constructed.

**Conclusion:** The crosstalk of hsa_circ_0000313-hsa-miR-6512-3p-*PEG10* and some related non-coding RNAs may take part in NAFLD’s pathogenesis, which could be the potential biomarkers of NAFLD in the future.

## Introduction

Currently, obesity takes center stage in a series of metabolic diseases. NAFLD is a manifestation of obesity in the liver. This general term describes a series of liver conditions ranging from steatosis to NASH, steatohepatitis with fibrosis, and cirrhosis. The worldwide incidence rate of NAFLD is increasing yearly. With a mean 25% prevalence globally, and the highest in the Middle East, 32% and the lowest in Africa, 14% (Younossi et al., 2016b). The overall medical costs have exceeded $100 billion every year in the United States (Mundi et al., 2020) and €35 billion in European countries (Younossi et al., 2016a).

In the past, the pathogenesis of NAFLD was mainly based on the “*two-hit*” hypothesis. The first hit is insulin resistance giving rise to the liver fat accumulation. The second hit is caused by comprehensive effects of mitochondrial dysfunction, inflammatory cytokines, lipid peroxidation, and oxidative stress due to the damage of hepatocytes and inflammatory response. However, there has been a growing number of recently researched ncRNAs in NAFLD (Gjorgjieva et al., 2019; Chien et al., 2020). Studies on genome-wide transcriptome have shown that a large number of ncRNAs, such as miRNAs and circRNAs, can regulate the expression of human genome. MiRNA is a kind of small single-stranded RNA that can inhibit the expression of its target genes. CircRNA is a class of endogenous ncRNA as well, different from the traditional linear RNA formed by reverse splicing which has plentiful miRNA binding sites and can act as miRNA sponge. It is a circular closed structure without a 5-terminal cap and 3-terminal tail (Chen and Yang, 2015).

What’s more critical, abnormal lipid metabolism in the liver is often accompanied by a disordered ncRNA expression (Singh et al., 2018). So far, the most accurate standard to diagnose NAFLD is liver biopsy. But for its invasiveness and expensiveness, it cannot be utilized widely. Non-invasive diagnostic methods should be considered for the continued investigation. In summary, the study on DEGs and ncRNA can explain the pathogenesis of NAFLD from another point of view. Also, it may be a non-invasive way of detection for NAFLD.

In our study, microarray data were obtained from the GEO database, and the DEGs were identified between individuals with or without NAFLD. Several databases predicted miRNAs and circRNAs targeting the DEGs to establish a circRNA-miRNA-mRNA network. In that case, some potentially therapeutic targets for NAFLD could be explored.

## Materials and Methods

### Information of Microarray Data

Microarray data of GSE63067 was downloaded from the GEO database^1^. Three groups of participants were included in this study. Two subjects were diagnosed with steatosis and nine with NASH, and the other seven healthy controls. The microarray platform was GPL570[HG-U133_Plus_2] (Affymetrix Human Genome U133 Plus 2.0 Array).

### Screening of DEGs

Using the online tool GEO2R/R package limma, DEGs were screened from the microarray by the cut-off point of *P*-value < 0.05 and | logFC| > 0.5. In this way, DEGs could be screened from the steatosis vs. healthy control, so did the non-alcoholic steatohepatitis vs. the control group. Only the overlapping genes in both of these groups could be selected as significant DEGs. Gene without a name should be excluded.

### Functional Enrichment Analysis of DEGs

The selected significant DEGs were uploaded to the Database for Annotation, Visualization, and Integrated Discovery (DAVID) version 6.8 Beta^2^ for further analysis. In this study, we were committed to studying the GO annotation and KEGG pathways of DEGs in the DAVID database. *P*-value < 0.05 was chosen as the threshold.

### Protein-Protein Interaction Network

Protein-protein interaction (PPI) networks for the significant DEGs were constructed by the Search Tool for the Retrieval of Interacting Genes database (STRING version 11.0)^3^ (Szklarczyk et al., 2011). The minimum required interaction score was 0.7. In this network, we hid the nodes without connections with others. Cytoscape3.7.2 (Doncheva et al., 2019)^4^ was applied to display the relationship between proteins. The hub gene of PPI network can be analyzed by cytohubba (Chin et al., 2014) in Cytoscape.

### CircRNA-miRNA-mRNA Network Construction

We chose the top 10 differential genes for further analysis. MiRWalk^5^, miRDB^6^, and miRNet^7^ databases were used to predict miRNA-targeted mRNA. The miRNA only in more than two of these databases would be retained. Starbase^8^ database was applied to select the miRNA and their targeted circRNA. Finally, the network of circRNA-miRNA-mRNA was constructed.

### Expression Validation of circRNA

Certification of the expression of circRNA was undertaken by using another GEO dataset (GSE134146). The cut-off point of *P*-value and logFC were the same with the DEGs’ selection standard.

## Results

### Analysis of DEGs in Liver Sample With or Without NAFLD

**FIGURE 1:**
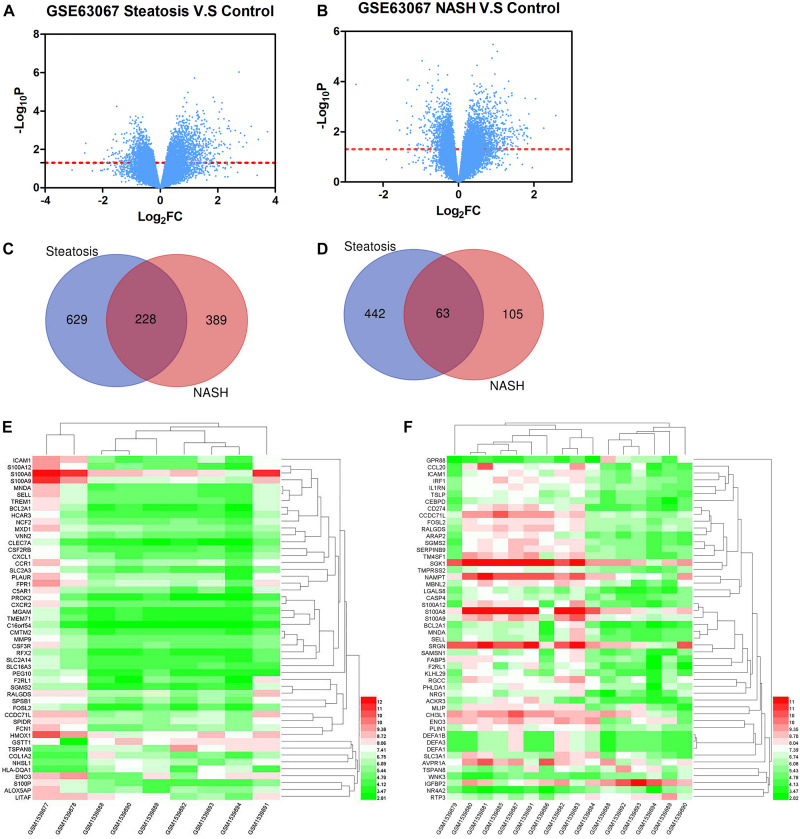
**(A)** The expression of all genes in steatosis vs. Control. **(B)** The expression of all genes in NASH vs. Control. **(C)** Venn diagram of upregulated genes in steatosis and NASH. **(D)** Venn diagram of downregulated genes in steatosis and NASH. **(E)** Top 50 differential genes in steatosis vs. Control. **(F)** Top 50 differential genes in NASH vs. Control.

Firstly, the data of GSE63067 was normalized. The expression of all genes in steatosis vs. Control (Figure 1A) and NASH vs. Control (Figure 1B) were exhibited in the volcano plot. There are 1362 differential genes in steatosis vs. Control: 857 upregulated genes and 505 downregulated genes. Simultaneously, 785 genes were found in NASH vs. Control: 617 upregulated genes and 168 downregulated genes. A Venn diagram was used to find the common genes in both groups: 228 upregulated genes (Figure 1C) and 63 downregulated genes (Figure 1D). The top 10 differential genes were shown in Table 1. Heatmap was used to indicate the top 50 differential genes in steatosis vs. Control (Figure 1E) and NASH vs. Control (Figure 1F).

**TABLE 1:** Top 10 differential genes in both steatosis vs. Control and NASH vs. Control.

**Gene symbol**	**Gene**	**LogFC**	***P*-value**
ICAM1	intercellular adhesion molecule 1	3.73	1.19E-03
S100A12	S100 calcium binding protein A12	3.42	3.02E-03
S100A9	S100 calcium binding protein A9	3.17	6.31E-04
S100A8	S100 calcium binding protein A8	3.00	4.45E-03
MNDA	myeloid cell nuclear differentiation antigen	2.93	1.88E-03
BCL2A1	BCL2 related protein A1	2.88	7.28E-03
SELL	selectin L	2.79	1.42E-03
PEG10	paternally expressed 10	2.74	9.31E-07
GPR88	G protein-coupled receptor 88	–2.72	1.30E-04
F2RL1	F2R like trypsin receptor 1	2.66	4.69E-02

### Analysis of Signaling Pathway and Biological Functions for 228 Upregulated DEGs by DAVID Database

**FIGURE 2:**
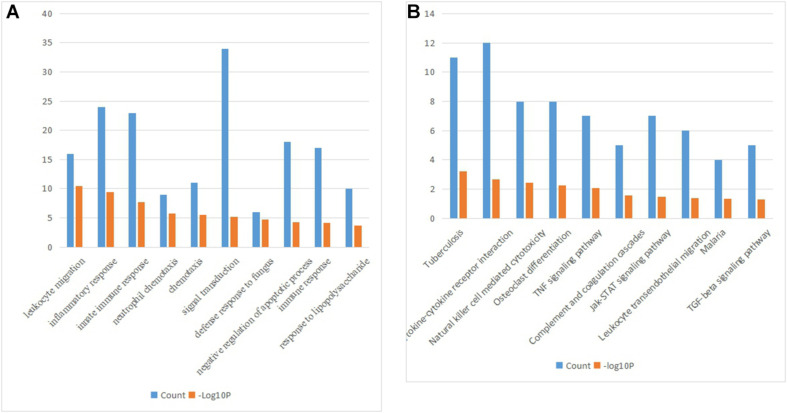
**(A)** The biological processes of the 228 common upregulated genes. **(B)** KEGG pathways of the 228 common upregulated genes.

The biological processes of the 228 common upregulated genes were analyzed by the DAVID database (Figure 2A). The major biological processes were included as followed: leukocyte migration (GO:0050900, *P* = 3.07E-11), inflammatory response (GO:0006954, *P* = 3.82E-10), innate immune response (GO:0045087, *P* = 2.16E-08), neutrophil chemotaxis (GO: 0030593, *P* = 1.56E-06), chemotaxis (GO:0006935, *P* = 2.95E-06), signal transduction (GO:0007165, *P* = 6.90E-06), defense response to fungus (GO:0050832, *P* = 1.84E-05), negative regulation of apoptotic process (GO:0043066, *P* = 5.73E-05), immune response (GO:0006955, *P* = 7.76E-05), response to lipopolysaccharide (GO:0032496, *P* = 2.16E-04). The crucial signaling pathways were exhibited in Figure 2B.

### Protein-Protein Network Construction and Hub Genes Analysis

**FIGURE 3:**
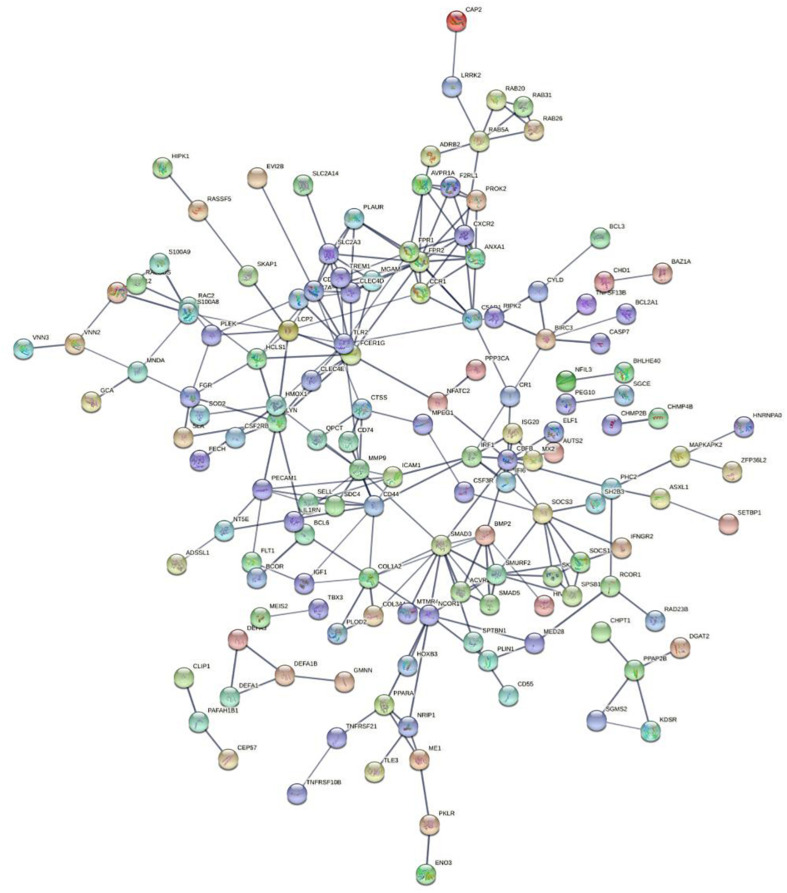
Protein-protein networks.

**FIGURE 4:**
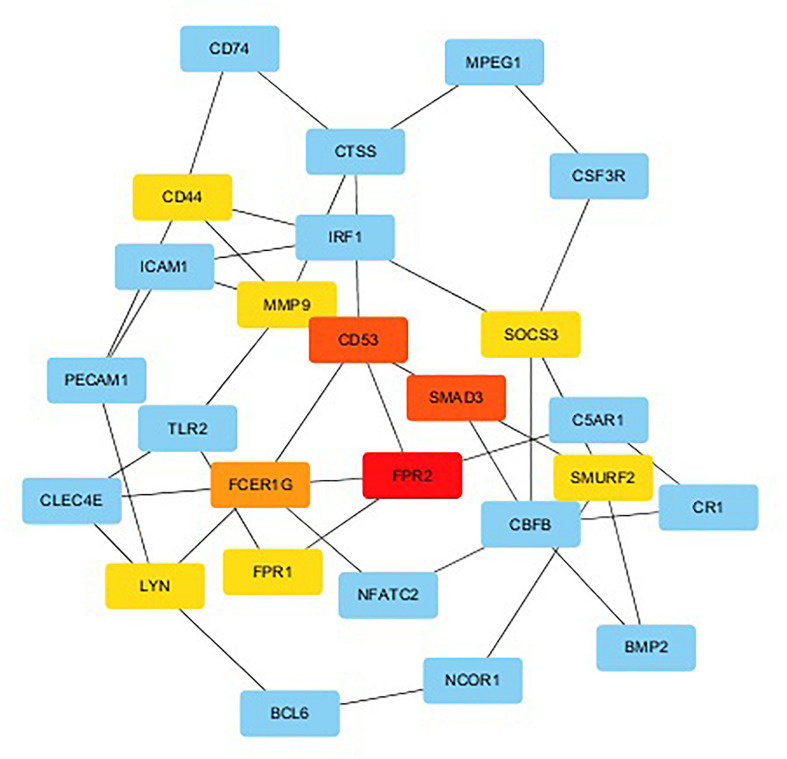
The top 10 hub genes and expanded subnetwork.

We used String to construct proteins’ interaction and selected the network’s essential genes (Figure 3). The top 10 of hub genes were *FPR2, SMAD3, CD53, FCER1G, SMURF2, FPR1, LYN, SOCS3, CD44, MMP9* (Figure 4). The score of each hub gene was shown in Table 2.

**TABLE 2:** The top 10 scores of hub genes.

**Gene symbol**	**Score**
FPR2	14
SMAD3	11
CD53	11
FCER1G	10
SMURF2	9
FPR1	9
LYN	9
SOCS3	9
CD44	9
MMP9	9

### Prediction of Target miRNA and circRNA

For those top 10 differential genes, we used databases (MiRWalk, miRDB, and miRNet) to forecast their target miRNAs. We found out one hundred thirty-nine miRNAs targeted these ten genes since one gene may target several miRNAs. For instance, gene GPR88 had five miRNAs as the potential targets, as miR-5591-5p, miR-181a-5p, miR-6507-5p, miR-920, and miR-628-5p. Meanwhile, several genes could target one miRNA. For example, miR-122-5p and miR-5004-5p could be targeted by more than one gene. Then we use the Starbase to predict the corresponding circRNAs for each of these 139 miRNAs. As a result, 902 circRNAs could be relevant to these miRNAs.

### Validation for Expression of circRNA in GSE134146

The selection criteria screened 752 differential circRNAs with 172 lowly expressed circRNAs and 580 highly expressed circRNAs in NAFLD. We took the interaction of these differential circRNAs with previous circRNAs. In the end, hsa_circ_0001453 and hsa_circ_0000313 could satisfy the above conditions. We could construct two circRNA-miRNA-mRNA as followed: hsa_circ_0001453-hsa-miR-27b-3p-*PEG10* and hsa_circ_0000313-hsa-miR-6512-3p-*PEG1*0.

## Discussion

In the past few years, overwhelming evidence has demonstrated that non-coding RNA may play a vital role in NAFLD progression. The Burgeoning high-throughput sequencing technologies make it possible for people to have a better understanding of non-coding transcripts. Starting from the differential genes on NAFLD, we speculated the miRNA and circRNA by online databases that they might bind to construct a circRNA-miRNA-mRNA interaction network to mirror NAFLD’s molecular mechanism.

Two hundred ninety-one differential genes were obtained from the GSE63067. The most differential genes were *ICAM1, S100A12, S100A9, S100A8, MNDA, BCL2A1, SELL, PEG10, GPR88*, and *F2RL1. FPR2, SMAD3, CD53, FCER1G, SMURF2, FPR1, LYN, SOCS3, CD44*, and *MMP9* were the hub genes in the protein-protein network, which indicated a leading role in predicting the risk of NAFLD. *PEG10* was the only left differentially gene in the circRNA-miRNA-mRNA network after multiple filters among all of these genes.

Some of these genes could directly affect NAFLD development, and the others may influence NAFLD by some related effects. The level of *ICAM1* was markedly enhanced after the stimulation of lipopolysaccharide in the liver, and the blockade of it may destroy the cells’ adhesion and expansion (Meng et al., 2019). *CD44*, is proved to be of great significance in non-alcoholic steatohepatitis. Patouraux found that *CD44* deficiency was strongly relevant to the activation of macrophages by lipopolysaccharide (LPS), hepatocyte damage-associated molecular patterns (DAMPs) and saturated fatty acids (Patouraux et al., 2017). *SMURF2*, a kind of E3 ubiquitin ligase, is able to attenuate liver fibrosis by inhibiting the hepatic stellate cell activation (Cai et al., 2018). These genes were closely connected with NAFLD (Chikada et al., 2020).

Additionally, apoptosis, inflammation, and insulin resistance were the critical processes in the pathogenesis of NAFLD. Inflammation is an inducing factor that can fuel the transition from steatosis to NASH (Schuster et al., 2018). Our study also discovered that inflammation attached great importance to NAFLD by analyzing the pathway and biological function of differential genes, which coincided with the previous studies. FPR are N-formylpeptide receptors involved in some pathogenic processes. Both *FPR1* and *FPR2* were found to have a relationship with NAFLD through influencing the inflammatory effect. Recently, a study indicated that *FPR2* deficiency can alleviate diet-induced insulin resistance, which is by weight loss and inhibiting inflammation (Chen et al., 2019). Some immune cells could also participate in many inflammatory processes. *S100A8, S100A9, and S100A12* were Ca^2+^ binding proteins that were abundant in many immune cells. They can accelerate immune cells to release inflammatory factors to destroy the immunity homeostasis (Wang et al., 2018). A recent study indicated that silencing *S100A8* could alleviate inflammation and oxidative stress, along with the changes of corresponding proteins (Hu and Lin, 2021). As for *PEG10*, it was closely related to adipocyte differentiation (Hishida et al., 2007). The expression of *PEG10* was correlated positively with insulin resistance and physical activity in NASH (Arendt et al., 2019). It is worth studying the left genes which have not been found a relationship with NAFLD in the previous research. By analyzing the biological processes of these differential genes, we discovered that inflammation might be of great importance in NAFLD’s pathogenesis. This conclusion was the same as the previous discoveries.

A great number of researches on differential genes of NAFLD have been heatedly discussed before. A study by Frades integrated genomic signatures of hepatocellular carcinoma derived from NAFLD (Frades et al., 2015). Compared with our study, the results they got integrate both human and mouse samples which maybe more universal. Another research by Feng et al. (2020) also used the GSE63067 to identify a total of 249 DEGs and one key gene (CCL20) for NAFLD. The DEGs they got may not be consistent with us because of the different screening criteria and grouping method. It is well known that NAFLD is comprised of four different conditions. In our study, we screened the DEGs in steatosis as well as in NASH, which makes the result more convinced. In terms of the miRNAs, we found miR-122-5p and miR-5004 had more than one target gene. MiR-122 is one of the most expressed MiRNAs in the liver (Yamada et al., 2015; Jampoka et al., 2018). In the different stages of NAFLD, the expression of miR-122 was different. *In vitro* and *Vivo* NAFLD model, miR-122 could promote the hepatic lipogenesis via suppressing the expression of *Sirt1* (Long et al., 2019). Reduced miR-122’s expression can lighten the fatty deposits and inflammation (Hu et al., 2019). In the longitudinal evaluation of one patient from NAFLD’s diagnosis until HCC, the expression of miR-122 may have a tendency to decrease before the progression of the fibrosis stage (Akuta et al., 2016). In our study, we speculated that miR-122-5p was downregulated for its opposite expression to the target gene. We summarized the possible reasons as followed. Firstly, we only chose one microarray to analyze the outcome. And it may have some incidental factors. Secondly, miRNA coming from a different source of samples may have an extra level of expression. Finally, the major subjects in this microarray were diagnosed with NASH. The miR-27-5p reported that miR-27-5p could increase lipid and TG contents (Murata et al., 2019) and it was an important adipogenic factor that can regulate adipogenesis in hyperlipidemia (Hsu et al., 2017). Still, we got the opposite outcome for the same reason with miR-122-5p. No existing links were found in miR-5004 and miR-6512-3p with NAFLD. As a result, miR-5004 and miR-6512-3p deserve further investigation which may be the potential biomarkers for NAFLD. There is no thorough research for these two circRNAs, so that we couldn’t validate our conjecture with previous research. All the results we got were based on the theoretical analysis. More experiments should be applied to verify these speculations.

In conclusion, we got some differential genes and constructed the hsa_circ_0000313-hsa-miR-6512-3p-*PEG10* network in NAFLD, which may be the underlying targets in diagnosis and treatment for NAFLD.

## Data Availability Statement

The datasets presented in this study can be found in online repositories. The names of the repository/repositories and accession number(s) can be found in the article/supplementary material.

## Author Contributions

CD and LS contributed to the conception and design of the study, the data analysis, the data interpretation, the manuscript drafting, and the critical revision of the manuscript. ZM, JD, and SJ contributed to the data analysis, the data interpretation, the manuscript drafting, and the critical revision of the manuscript.

## Conflict of Interest

The authors declare that the research was conducted in the absence of any commercial or financial relationships that could be construed as a potential conflict of interest.

## Footnotes

1 www.ncbi.nlm.nih.gov/geo/

2 https://david-d.ncifcrf.gov/

3 https://string-db.org/

4 http://www.cytoscape.org/

5 http://mirwalk.umm.uni-heidelberg.de/

6 http://www.mirdb.org/

7 https://www.mirnet.ca/miRNet/home.xhtml

8 http://starbase.sysu.edu.cn/
